# Research Progress on the Pharmacological Action of Schisantherin A

**DOI:** 10.1155/2022/6420865

**Published:** 2022-02-12

**Authors:** Zehao Xiao, Wen Xiao, Guilin Li

**Affiliations:** ^1^Queen Mary School, Medical College of Nanchang University, Nanchang 330006, China; ^2^Department of Pathology, Medical College of Nanchang University, Nanchang 330006, China; ^3^Department of Physiology, Medical College of Nanchang University, Nanchang 330006, China

## Abstract

Schisantherin A (Sch A) is a dibenzocyclooctadiene lignan monomer isolated from the fruit of *Schisandra chinensis* (Turcz.) Baill. (*S. chinensis*). At present, many studies have shown that Sch A has a wide range of pharmacological effects, including its anti-Parkinson and anti-inflammatory effects and ability to protect the liver, protect against ischemia-reperfusion (I/R) injury, suppress osteoclast formation, and improve learning and memory. Its mechanism may be related to the antioxidant, anti-inflammatory, and antiapoptotic properties of Sch A through the MAPK, NF-*κ*B, AKT/GSK3*β*, and PI3K/AKT pathways. This is the first review of the recent studies on the pharmacological mechanism of Sch A.

## 1. Introduction

Natural products play a very important role in drug discovery and development. Many kinds of small-molecule drugs have been isolated from plant sources. *Schisandra chinensis* (Turcz.) Baill. (*S. chi*nensis) is a traditional Chinese medicine (TCM) in the Angiosperm Phylogeny Group (APG)-II system, in which Schisandraceae and Magnoliaceae are placed in the same category as Austrobaileyales. *S. chinensis* has hepatoprotective, neuroprotective, anti-inflammatory, antiviral, antioxidative, anticancer, detoxification, antiapoptosis, and immunostimulant activities [1–4]. *S. chinensis* has been used in the treatment of many diseases, such as gastrointestinal diseases, cardiovascular diseases, bodily fatigue and weakness, respiratory failure, excessive sweating, and insomnia [2, 5]. Clinical studies over the last ten years have indicated that *S. chinensis* can act on the nervous system, reduce fatigue, and treat palpitation and hot flashes [3, 6], and *S. chinensis* was suggested to be used to improve and treat cognitive impairment in models of neurodegenerative diseases and Alzheimer's disease [7]. The ability to resist oxidative stress in the liver is also evident. *S. Chinensis* can significantly improve the antioxidant capacity of the body, reduce oxidative active substances, and effectively improve liver function in patients [8–10]. *S. chinensis* can also be used in combination with tacrolimus to protect the liver and reduce tacrolimus requirements in the treatment of transplant patients [11, 12]. Additionally, clinical studies have shown that the extract of *S. chinensis* can be used as a health supplement, has beneficial effects on muscle strength and lactic acid levels [13], and can also be used to regulate the composition of the intestinal flora [14].

**Figure 1:**
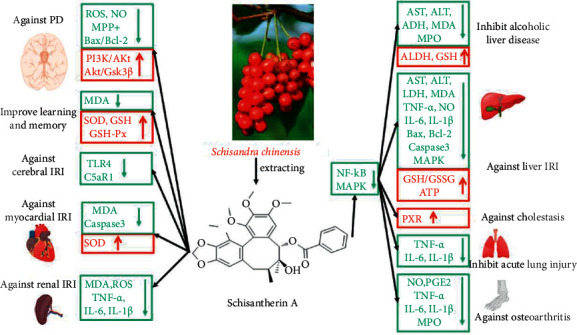
Chemical structural formula and pharmacological effects of schisantherin A.

The biological activity and pharmacological use of *S. chinensis* are related to dibenzocyclooctadiene lignans. Schisantherin A (Sch A), a dibenzocyclooctadiene lignan monomer and primary biologically active lignan also known as gomisin C, schizandrin A, and wuweizi ester A, is isolated from the fruit of *S. chinensis*. Its molecular weight is 536.57 g/mol, its molecular formula is C_30_H_32_O_9_, and its chemical structure is shown in Figure 1. Sch A is highly soluble in benzene, chloroform, and acetone; soluble in methanol and ethanol; and insoluble in petroleum ether and water. The structure of Sch A contains a methylenedioxy group, methyl groups, methoxy groups, a benzoyloxy group, and a hydroxyl group. Therefore, the loss of C_2_H_2_O_2_, benzoic acid, H_2_O, methyl, and methoxy groups are the main and natural metabolic pathways.

Sch A has the highest content and the most effective advantage in the lignan extract of *S. chinensis* [15, 16]. In a 2019 study, the metabolic profiles of Sch A *in vitro* (rat liver microsomes) and *in vivo* (rat plasma, urine, and bile) were assessed by ultra-high-performance liquid chromatography-quadrupole time-of-flight tandem mass spectrometry (UPLC-Q-TOF-MS/MS) [17]. Sch A was found to be more complex *in vivo* than *in vitro* and undergo both hepatic and extrahepatic metabolic pathways in its biotransformation. The metabolic process mainly involves the loss of C_7_H_4_O and C_7_H_4_O_2_, as well as further oxidative transformation, carboxylic acid reaction, and the loss of CH_2_ and CH_2_O groups. Moreover, the coupling of Sch A with glucuronic acid, sulfate, taurine, glutathione, and glucose may also account for its multiple effects. This study showed that the methoxy group and biphenyl cyclooctene are the main metabolic sites in Sch A. This was also demonstrated in a trial in which the efficacy of Sch A and other major lignans in the treatment of Parkinson's disease (PD) was compared [16]. Sch A has a greater neuroprotective function than schizandrin A and schizandrol A, which have no methylenedioxy groups, and this activity may be enhanced by the addition of a benzoyloxy substituent on the cyclooctadiene ring. This may be because the benzoyloxy and methoxy groups in Sch A inhibit the production of 1-methyl-4-phenylpyridinium ion (MPP+), similar structures to which are also present in the antipsychotic drugs sulpiride and amishupiride [18].

### 1.1. Extraction Technology

Lignans are traditionally separated by similar procedures. Ground seeds are extracted with ethanol/water or by using heat reflux [19, 20] and Soxhlet [19]. At present, most Sch A is extracted by ethanol extraction and heating. However, the extraction efficiency is low due to the long heating time and the instability of Sch A under heat [19]. Additionally, these methods cannot avoid the problem of the excessive consumption of medicinal materials and are labor-intensive, time-consuming, and harmful to human health and the environment. Therefore, new extraction methods, such as microwave-assisted extraction (MAE) [21] and supercritical CO_2_ fluid extraction (SFE) [22], have been developed. However, due to the high cost of these technologies, which limits their large-scale application, the industrial applications of this technology are still limited. Currently, an extraction method consisting of an aqueous two-phase system coupled with ultrasound is also being used [15]. Three steps are needed: solid-liquid extraction, liquid-liquid microextraction, and ultrasonic extraction. The main principle of the application of ultrasonic extraction technology is the use of the ultrasonic cavitation effect to accelerate the dissolution of the active ingredients of plant cell membranes, allowing the solvent to enter the cells, releasing the medicinal compounds, and the secondary effect of the ultrasonic wave, such as that upon mechanical vibration, emulsification, diffusion, crushing, chemistry, and so on, can also accelerate the diffusion of the extract released with the solvent mixture. Compared with the traditional solvent extraction method, this method has the advantages of a shorter extraction time, no need for heating, good suitability for extracting substances, and a higher target components extraction rate, and it can reduce the required amount of solvent and is conducive to further refinement and purification. Under optimal conditions with a solvent-to-solid ratio of 20 : 1, ultrasonic power of 800 W, and extraction time of 61.1 min, 13.10 mg/g schizandrin, 1.87 mg/g Sch A, and 1.84 mg/g schizandrin could be extracted [15]. As a new extraction technology for TCM, ultrasonic extraction has good application prospects.

Recently, increasing amounts of data have shown that Sch A has broad pharmacological functions, including its antioxidant, sedative [23], anti-Parkinson, and anti-inflammatory effects and ability to protect the liver and protect against ischemia-reperfusion (I/R) injury, suppress osteoclast formation, and improve learning and memory. This summary aims to present the pharmacological effects and mechanisms of Sch A and provides a theoretical reference for future research.

## 2. The Role of Sch A in Diseases

### 2.1. Sch A against PD

PD, one of the most common neurodegenerative diseases, is characterized by degeneration of dopaminergic neurons in the substantia nigra with the formation of Lewy bodies. Previous studies have suggested that PD is a movement disorder as it mainly manifests by movement symptoms such as rigidity, trembling, and/or postural gait instability [24].

The pathological processes of PD include oxidative stress, impairment of the antioxidant response, mitochondrial dysfunction, and the accumulation of *α*-synuclein aggregates. These changes are known to aggravate damage to dopaminergic neurons [25].

In recent years, modern medical research has shown that the pathogenesis of PD is complex and involves multiple factors. Past experiments have shown that the pathological changes in PD involve not only dopaminergic neurons in the substantia nigra and striatum system but also involve other parts of the dopaminergic system and the 5-hydroxytryptamergic, norepinephrinergic, and cholinergic systems, as well as the dynorphin, enkephalin, and other peptide transmitter systems [26–28].

Sch A has the potential to become an oral drug for the treatment of PD and to provide a new way to improve the bioavailability of PD drugs. Sch A was reported to act against PD through *in vitro* and *in vivo* experiments. In a PD mouse model, Sch A has been widely used to protect against the loss of TH-positive dopaminergic neurons induced by 1-methyl-4-phenyl-1,2,3,6-tetrahydropyridine (MPTP). Among the five dibenzocyclooctadiene lignans of *Schisandra* (schizandrin A, schizandrin B, schizandrin C, schizandrol A, and Sch A), Sch A presented the strongest neuroprotective activity. This suggests that Sch A is a key component of dibenzocyclooctadiene lignans with neuroprotective activity. The results also indicate that unique positions and constituents on different chemical arms (including methylenedioxy, methoxy, and benzoyloxy groups) in the dibenzocyclooctadiene lignan may play an important role in the neuroprotective activity of Sch A. *In vitro* experiments showed that Sch A protected SH-SY5Y cells and significantly decreased the cytotoxicity induced by MPP+. The possible molecular mechanisms are related to two pathways: the phosphatidylinositol-4,5-bisphosphate 3-kinase (PI3K)/protein kinase B (Akt) signaling and cAMP-response-element-binding protein-mediated B-cell lymphoma 2 (Bcl2). It is well known that PI3K/Akt signaling promotes cell survival. Sch A could increase the phosphorylation level of Akt and rescue the decrease in Akt phosphorylation induced by MPP+. Furthermore, Sch A could improve cell viability, which was inhibited by MPP+, while Akt inhibitor IV, a PI3K/Akt inhibitor, could abolish the effect of Sch A. Thus, it can be inferred that the neuroprotective effects of Sch A are exerted through the PI3K/Akt pathway. The ratio of Bcl2-associated X (Bax)/Bcl2 is one of the major biomarkers used to evaluate MPP+-induced apoptosis. Sch A decreased the Bax/Bcl2 ratio by upregulating Bcl2 expression, which was reduced by MPP+. The inhibitory effect of Sch A on apoptosis was closely related to the phosphorylation of CREB upstream of Bcl2. Therefore, the neuroprotective effect of Sch A is also related to CREB-mediated Bcl2 [16].

To improve the poor water solubility of Sch A, Chen et al. formulated it as nanocrystals. They found that Sch A concentration was considerably increased in the brain after oral administration of the nanocrystals. Sch A nanocrystals remarkably improved swimming behavior defects induced by MPTP and significantly decreased the death of dopaminergic neurons and locomotion deficiency induced by MPTP in zebrafish. The authors also found that activation of the Akt/glycogen synthase kinase-3*β* (Gsk3*β*) pathway may be involved in the strong neuroprotective effects of Sch A [29].

In addition, many studies have provided evidence that the formulation of Sch A in nanoemulsion can significantly increase the absolute bioavailability of Sch A from 4.3% to 47.3% [30], and its formulation in small methoxy poly (ethylene glycol) (mPEG)-poly (lactic-co-glycolic acid) (PLGA) nanoparticles improved the bioactivity, oral bioavailability, cross-barrier transportation, and brain uptake of Sch A [31].

Other studies showed that Sch A protected neurons from toxicity induced by glutamate [32] and dopaminergic neuron damage and cytotoxicity induced by 6-hydroxydopamine (6-OHDA) [33] in zebrafish models and human SH-SY5Y neuroblastoma cells. In zebrafish, Sch A prevented 6-OHDA-induced dopaminergic neuron loss. In 6-OHDA-treated SH-SY5Y cells, Sch A improved intracellular reactive oxygen species (ROS) accumulation and reduced nitric oxide (NO) overproduction by inhibiting the expression of inducible nitric oxide synthase (iNOS). Sch A also repressed the activation of GSK3*β*, mitogen-activated protein kinases (MAPKs), and PI3K/Akt signaling pathway members [33].

### 2.2. Sch A Improves Learning and Memory

Over the past century, the treatment of disease has significantly increased life expectancy. However, age-related cognitive decline has become one of the greatest threats to the health of elderly individuals [34]. In the aging process, a decline in learning and memory is a prominent manifestation of aging of the central nervous system. Therefore, the development of drugs with auxiliary memory improvement functions will play an important role in improving memory and the quality of life of elderly individuals.

Sch A has been suggested to protect against oxidative stress, neurodegeneration, and cognitive impairment, and Sch A is a potential drug for the treatment of Alzheimer's disease [35]. Studies have shown that successive intracerebroventricular administration of Sch A (0.01 and 0.1 mg/kg) for 5 days notably improved A*β*1–42-induced cognitive deficits. Sch A increased spontaneous alternation behavior (examined by the Y-maze) and the number of avoidances (tested by the shuttle-box) and significantly reduced the escape latency (measured by the Morris water maze test). Additionally, Sch A restored the activities of superoxide dismutase (SOD) and glutathione peroxidase (GSH-Px) and the levels of A*β*1–42, glutathione (GSH), and malondialdehyde (MDA) in the hippocampus and cerebral cortex. These effects of Sch A might be related to its antioxidant capacity [36].

Sch A also drastically improved D-galactose-induced learning and memory impairment in mice. Furthermore, Sch A significantly improved learning and memory behavior. For example, Sch A increased the latency to enter a dark compartment, reduced the number of errors (examined by the step-through test) and shortened the time required to find the platform, and reduced the number of platform passes (as measured by the Morris water maze test). Sch A also increased SOD activity, decreased MDA content in both the hippocampus and peripheral blood of mice, and decreased the 8 hydroxy deoxyguanosine (8-OHdG) content in the hippocampus of the mice. Moreover, Sch A reduced the expression levels of p19, p53, and p21 and elevated the expression levels of cyclin D1 and CDK4 and retinoblastoma protein (Rb) phosphorylation in the hippocampus [37].

### 2.3. Anti-Inflammatory Effects of Sch A in Alcoholic Liver Disease, Acute Lung Injury, and Osteoarthritis

Long-term heavy drinking has been suggested to cause alcoholic liver disease. Many influencing factors, such as sex (women are more susceptible) [38], genetics [39], malnutrition, infection with hepatitis c virus (HCV) [40], obesity, and smoking [41], affect an alcoholic liver disease.

At present, Western medicine provides nutritional support, especially branched-chain amino acids, to treat alcoholic liver disease [42]. Other compounds have also been proven to be useful, such as glutathione, cysteine, tiopronin, metadoxine [43], S-adenosylmethionine, polyene phosphatidyl choline [5, 44], compound glycyrrhizin, silymarin [45], taurine [46, 47], and glucocorticoid, and if all else fails, liver transplantation is performed [48, 49]. However, their curative effect is not obvious, and many drugs have serious adverse reactions and complications.

In an alcohol-induced liver injury mouse model treated with Sch A, aspartate aminotransferase (AST), alcohol dehydrogenase, and alanine transaminase (ALT) activities and vesicular steatosis were significantly lower than those in the control group, suggesting that Sch A had alleviated alcohol-induced pathologic changes in the liver and improved liver function. Sch A decreased the activity of alcohol dehydrogenase (ADH), increased the activity of acetaldehyde dehydrogenase (ALDH), and reversed the metabolic imbalance induced by alcohol. The elevated MDA level and myeloperoxidase (MPO) activity and reduced activities of GSH activity induced by alcohol were strongly abrogated by the administration of Sch A. Sch A also suppressed the microsomal ethanol oxidizing system and alcohol-induced activation of the nuclear factor-kappa B (NF-*κ*B) pathway. The hepatoprotective effects of Sch A may be related to its regulation of alcohol metabolism and inhibition of the NF-*κ*B pathway [50].

The pathogenesis of acute lung injury (ALI) is attributed to an excessive inflammatory response leading to the destruction of lung tissue, which is manifested as tumor necrosis factor-necrosis alpha (TNF-*α*) induction by various cytokines, the increased expression of interleukin-1 (IL-1), and an inflammatory cascade mediated by cytokines, which aggravates lung tissue injury. Moreover, a more severe form of ALI called acute respiratory distress syndrome (ARDS) can occur. Therefore, inhibiting inflammatory overreaction is an effective way to prevent and treat ALI/ARDS [31, 51].

Many studies have shown the anti-inflammatory functions of Sch A. When mice with ALI and ARDS were treated with Sch A, the levels of TNF-*α*, IL-6, and NF-*κ*B decreased. Sch A may also play a role in treating ALI/ARDS by blocking the NF-*κ*B pathway [52, 53].

Osteoarthritis (OA), a chronic degenerative disease characterized by articular cartilage destruction, synovial membrane inflammation, joint space narrowing, osteophytosis, and subchondral bone remodeling with joint pain, swelling, deformity, and movement disorder, is often found in weight-bearing joints such as the knees and hips [54–56].

The onset of OA is closely related to imbalance between the synthesis and degradation of the extracellular matrix in joints caused by the accumulation of senescent chondrocytes, the degradation of articular cartilage, and persistent pathological inflammation [57]. Therefore, inhibiting the inflammatory response in OA and protecting the integrity of articular cartilage may improve the pathological changes that occur in OA and relieve the pain of OA patients. Sch A exerted a dose-dependent anti-inflammatory effect by inhibiting the production of NO, prostaglandin E2 (PGE2), and TNF-*α* induced by IL-1*β* in human OA chondrocytes [58]. In a lipopolysaccharide (LPS)-induced ARDS mouse model, pretreatment with Sch A significantly improved LPS-induced histopathological changes and reduced the levels of IL-1*β*, IL-6, and TNF-*α* in bronchoalveolar lavage fluid [53]. The release of proinflammatory cytokines affects the expression of various molecules and eventually leads to neutrophil aggregation and lymphocyte activation [59–61]. The reduction in neutrophil infiltration in an MPO immunofluorescence assay further confirmed the anti-inflammatory activity of Sch A.

### 2.4. Sch A against Cholestasis

Cholestasis is a deficiency of bile acid formation and flow, leading to residual toxic bile acids in the liver. Intrahepatic cholestasis can cause hepatobiliary damage due to the accumulation of excess bile acids throughout the body and liver. Without proper treatment, long-term cholestasis can cause symptoms such as itching, osteoporosis, fatigue, jaundice, and hypercholesterolemia and lead to liver fibrosis and cirrhosis [62–64]. Thus, the treatment of cholestasis urgently needs to be improved.

Ursodeoxycholic acid (UDCA), an anti-inflammatory agent that reduces bile salt toxicity, is the only Food and Drug Administration (FDA)-approved treatment for cholestasis, but UDCA is not used for all patients, as it can cause severe abnormal alkaline phosphatase levels in some cases, which may lead to death [65–67].

Moreover, obeticholic acid has the potential to treat cholestasis. On the one hand, obeticholic acid improved cholestasis by decreasing the alkaline phosphatase content and total bilirubin level, but on the other hand, it was more prone to pruritus compared with placebo [68]. Therefore, it is necessary to develop new drugs with fewer adverse events as soon as possible.

An interesting study has shown that *Schisandra sphenanthera,* including Sch A, can activate pregnane X receptor (PXR) in mice, which increases the expression of UDP glucuronosyltransferase family 1 member A1 (UGT1A1) and the cytochrome P450 (CYP) family members CYP2B10 and CYP3A11 [69]. As a bile acid homeostasis regulator, PXR can modulate metabolism (Cyp3a11, Ugt1a1), transport (Oatp2, Mrp3), biosynthesis (Cyp7a1), and the excretion of xenobiotics and bile acids *in vivo* [70–72]. In addition, a dual-luciferase reporter gene assay was used to study the transactivation effect of lignans on the human pregnane X receptor (hPXR), and the results showed a similar effect [73]. These analyses revealed that Sch A can protect against lithocholic acid (LCA)-induced cholestatic liver injury.

### 2.5. Sch A against I/R Injury

I/R injury, which consists of two stages, ischemia and reperfusion injury, occurs after the restoration of oxygen delivery to a hypoxic organ [74, 75]. The liver is one of the organs most injured by I/R [76]. To illustrate, in the early stage of hepatic I/R, due to increased blood viscosity, a large number of white blood cells activate and block blood vessels, accompanied by vasoconstriction, and endothelial cell swelling, microcirculation disorders, and cell ischemia and hypoxia are further aggravated [74, 77, 78]. Eventually, this can lead to liver failure and multiple organ dysfunction syndromes (MODS) [79].

The protective effect of Sch A against hepatic I/R injury in mice was studied. After hepatic reperfusion, Sch A pretreatment remarkably restored liver function, as exhibited by limited histological damage; decreased levels of serum ALT, AST, and lactate dehydrogenase (LDH); and reduced neutrophil infiltration. Pretreatment with Sch A attenuated the expression of proinflammatory cytokines, such as IL-1*β*, IL-6, and TNF-*α*, in an I/R-induced inflammatory state. The results showed that the protective effect of Sch A was related to a decrease in oxidative/nitrosative stress, as characterized by a decrease in MDA production and total NO content and a high GSH/glutathione disulfide (GSSG) ratio. Moreover, ischemic mice pretreated with Sch A showed a similar increase in adenosine triphosphate (ATP) content in the liver. These findings suggest that Sch A improves mitochondrial function in hepatocytes by alleviating oxidative/nitrosative stress. Sch A pretreatment not only reduced I/R-induced oxidative stress and inflammation but also remarkably decreased hepatocyte apoptosis, which was evident in the diminished number of terminal deoxynucleotidyl-transferase-mediated dUTP Nick end-labeling (TUNEL)-positive hepatocytes and reductions in the apoptosis-related proteins Bax, Bcl2, and cleaved caspase-3. Sch A pretreatment reversed the upregulated phosphorylation of c-Jun N-terminal kinase (JNK), p38, and extracellular regulated protein kinases (ERKs) induced by hepatic I/R injury, suggesting that Sch A plays a role in protecting against I/R-induced liver injury by hindering activation of the MAPK pathway. Therefore, Sch A pretreatment is a promising method for hepatic protection in I/R-induced pathological environments, such as blood occlusion during liver resection and liver transplantation [80]. Ischemic heart disease is one of the most common cardiovascular diseases in clinical practice. Reperfusion of ischemic coronary arteries can effectively reduce total mortality [81]; however, myocardial ischemia-reperfusion injury (MIRI) occurs after coronary reperfusion. MIRI includes microvascular injury, arrhythmia, reversible cardiac dysfunction, and myocyte apoptosis. Various mechanisms that include reperfusion injury, intracellular Ca^2+^ overload, oxidative stress, and inflammatory factors are involved in changes in myocardial structure and function [82].

For the treatment of I/R injury, compared with the Western medicine reperfusion therapy, which exhibits limitations, the advantages of TCM are particularly prominent; for instance, TCM supports the use of multiple approaches and multiple targets. TCM compounds and related ingredients can alleviate MIRI injury through a variety of mechanisms, and remote ischemic treatment may have a higher clinical value than ischemic preconditioning (IPC) and ischemic postconditioning (IPOC) [83, 84]. Chang et al. studied the protective effect of Sch A against MIRI. The results showed that Sch A has a strong protective effect on cardiac function by reducing the left ventricular systolic pressure (LVSP), left ventricular end-diastolic pressure (LVEDP), and occurrence of arrhythmia. Furthermore, Sch A significantly reduced the infarction area and release of MDA. However, SOD activity increased, and the changes in myocardial histopathology were significantly alleviated, showing the strong protective effect of Sch A against myocardial injury. In addition, Sch A could significantly reduce the apoptosis of myocardial cells and caspase-3 activity, showing a strong protective effect. These results illustrate that Sch A has a protective effect against MIRI [85].

Inflammation and oxidative stress are key to acute and long-term nerve tissue injury after cerebral ischemic stroke [86]. After cerebral ischemic stroke, blood flow in the local brain tissue is reduced, and disorder in the tissue blood supply leads to injury and the death of neurons at the damaged site and in the cerebral cortex, as well as the impairment of synaptic structure and conduction function [87]. Timely repair of the damaged neuronal structure and prevention of the loss of neuronal function is important for the treatment of this disease. Studies have shown that both autophagy and apoptosis signals in neurons can be activated after cerebral I/R. Excessive cellular autophagy will cause cells to undergo autophagic death [88], while moderate autophagy activation is conducive to the survival of neurons and induces neuroprotection [89].

Sch A may be an effective neuroprotective agent with antioxidant, anti-inflammatory, and antiapoptotic effects on ischemia and related diseases. Shi et al. assessed the protective effect of SCA on cerebral I/R injury and explored the mechanisms related to Toll-like receptor 4 (TLR4) and complement C5a receptor (C5aR1, CD88) signaling pathways *in vitro* and *in vivo*. The results showed the potent neuroprotective effects of Sch A, which reduced oxygen and glucose deprivation and the reperfusion injury-induced apoptosis of primary cultured cortical neurons. It restored homeostasis through different features, such as inflammation, oxidative stress, and apoptosis, by inhibiting the TLR4 and C5aR1 signaling pathways [90]. Furthermore, the activation of C5aR1, a G-protein-coupled receptor highly expressed in neutrophils, can aggravate acute and chronic inflammation [91]. The NF-*κ*B signaling pathway can be activated by TLR4 and TNFR1, which induces high cytokine expression levels [92, 93].

With the increasing incidence of kidney transplantation and partial nephrectomy, renal I/R injury is difficult to avoid. Blood reperfusion, the main factor leading to kidney injury, can cause acute tubular necrosis, induce renal failure, affect the recovery of renal function after surgery, and in severe cases endanger patient survival [94]. Gong and Wang examined the effect of Sch A on renal I/R injury *in vitro*. Sch A decreased the levels of MDA and ROS and reduced the production of TNF-*α*, IL-1*β*, and IL-6 in a hypoxia/reoxygenation-stimulated human renal tubular epithelial cell line (HK-2). Moreover, Sch A suppressed I/R-induced HK-2 cell apoptosis through the PI3K/Akt signaling pathway [95], which is part of the casein kinase signaling pathway and involved in cell proliferation, angiogenesis, and other activities through its effects on a variety of intracellular second messengers and participation in several important steps in the process of I/R injury [96]. These experiments showed the potent protective effects of Sch A against renal I/R injury.

## 3. Summary and Prospects

Sch A has primary anti-inflammatory, antioxidant, and antiapoptotic properties (Figure 1). It is mainly involved in the MAPK, NF-*κ*B, AKT/GSK3*β*, and PI3K/AKT pathways, reducing oxidative damage by reducing the production of MDA and ROS, and reducing inflammation by reducing IL-1*β* and TNF-*α* (Table 1). This may be due to the methoxy group and the biphenyl cyclooctene in its structure, which effectively improve the AKT pathway and reduce MPP+ and protect nerves. In addition, metabolic derivatives of Sch A can be coupled with glucuronic acid, sulfate, taurine, glutathione, and glucose to produce possible anti-inflammatory and antioxidant effects.

Our understanding of Sch A is still limited, and the specific mechanism by which Sch A affects signaling pathways and genes is still unknown, even though some progress has been made. Many derivatives of Sch A have been obtained by chemical synthesis, but their pharmacological activities also need further study. To obtain the maximum advantages of TCM, understanding the interaction between different components of *S. chinensis* is also indispensable. With further research, it is hoped that Sch A and its derivatives can become new clinical drugs due to their characteristics, which include their high efficiency, low adverse reactions, and easy access.

I/R: ischemia/reperfusion; MCAO/R: middle cerebral artery occlusion and reperfusion.

## Figures and Tables

**Table 1:** Summary table for disease, study model, route of administration, dose, related signaling pathways, genes, and therapeutics outcome of Sch A.

Disease	Study model	Route of administration	Dose	Pathways and related genes	Therapeutics outcome	References
Parkinson's disease	Human neuroblastoma SH-SY5Y cells	Medium	3–25 *μ*M	PI3K/Akt	Protect against MPP+-induced cell damage in a concentration-dependent manner	[16]
	Male C57BL/6 mice	Intragastric gavage	30, 100, 300 mg/kg/d, 14 d			
	Male SD mice	Intragastric gavage	SA-NC 4 mg/kg		Increase SA oral absorption	[29]
	Zebrafish embryo	Embryo medium	SA-NC 1, 3, 10 *μ*M		Attenuated MPTP-induced neurotoxicity on zebrafish	
	Human neuroblastoma SH-SY5Y	Medium	SA-NC 30 *μ*M	Akt/Gsk3*β*	Blocked the MPP+-induced repression of p-Gsk3*β*	
	Cortical cells of fetal SD mice	Medium	0.1, 1.0, 5.0 *μ*M		Possess therapeutic potential against oxidative neuronal damage induced by excitotoxins	[32]
	Human neuroblastoma SH-SY5Y	Medium	3, 6, 12, 25, 50, 100 *μ*g/ml	ROS/NO AKT/GSK3*β*		[33]

Alzheimer's disease	A*β*1–42-induced learning and memory impairment in male 12-week-old KM mice	Intragastric gavage	0.01, 0.1 mg/kg for 5 days		Protect against cognitive deficits, oxidative stress, and neurodegeneration	[24, 36]
	D-galactose-induced learning and memory impairment in male ICR mice	Orally	1.25, 2.50, and 5.00 mg/kg for 42 days.	p19/p53/p21/cyclinD1/CDK4 genes	Antioxidation	[26, 37]

Alcohol-induced liver injury	Alcohol-containing diet-induced liver injury in eight-week-old male C57BL/6 mice	Oral gavage	100 mg/kg and 200 mg/kg	NF-*κ*B	Anti-inflammation and antioxidation	[39, 50]
Acute lung injury	Lipopolysaccharide-induced acute lung injury in male BALB/c mice	Intragastric gavage	25, 50 mg/kg, and 40 mg/kg	NF-*κ*B and MAPK	Anti-inflammation	[41, 42, 52, 53]
Osteoarthritis	Primary chondrocytes from articular cartilage samples	Medium	9.5, 19, 38 *μ*M	NF-*κ*B and MAPK	Anti-inflammation	[47, 58]
Cholestasis	Lithocholic acid-induced cholestasis in adult male C57BL/6J mice	Oral gavage	100 mg/kg,	hPXR-regulate genes, CYP3A4, UGT1A1 and OATP2.	Hepatoprotection and detoxification and efflux of toxic bile acids	[58, 69]
Liver ischemia-reperfusion injury	70% hepatic warm ischemia was used in male C57BL/6 mice	Gavage	200 mg/kg/day gavage for 5 days,	MAPK	Anti-inflammation, antioxidation against apoptosis	[69, 80]
Myocardial ischemia-reperfusion injury (MIRI)	MIRI in male Wistar rats by occluding the left coronary artery for 45 min followed by 2 h of reperfusion	Microemulsion injection	40 mmol/kg	Bcl2/Bax/Caspase-3	Inhibiting cardiomyocyte apoptosis	[74, 85]
Cerebral ischemic stroke	I/R-induced neuronal injury in SD rat embryos in the hypoxic chamber	Medium	1.25, 2.5, and 5 *μ*g/ml	TLR4 and C5aR1	Alleviated neurological deficits, reduced infarct volume, and attenuated oxidation stress, inflammation, and apoptosis in the ischemic parietal cortex of rats after MCAO/R injury	[79, 90]
Acute kidney injury	Hypoxia/reoxygenation injury in renal tubular epithelial cells by incubating in the hypoxic chamber	Medium	5, 10, 20 *μ*M	PI3K/Akt	Attenuated oxidation stress, cell apoptosis, and inflammation	[84, 95]
Ulcerative colitis	HCT116 cells	Medium	1 *μ*g/ml		Inhibits H_2_O_2_-induced apoptotic cell death in intestinal epithelial cells	[97]

## Abbreviation

ROS: Reactive oxygen species
NO: Nitric oxide
MPP+: 1-methyl-4-phenylpyridinium ion
Bax: Bcl2-associated X
Bcl2: B-cell lymphoma 2
PI3K: Phosphatidylinositol-4,5-bisphosphate 3-kinase
Akt: Protein kinase B
Gsk3*β*: Glycogen synthase kinase-3*β*
MDA: Malondialdehyde
SOD: Superoxide dismutase
GSH: Glutathione
GSH-Px: Glutathione peroxidase
TLR4: Toll-like receptor 4
C5aR1: Complement C5a receptor
IRI: Ischemia-reperfusion injury
TNF-*α*: Tumor necrosis factor-alpha
IL-6: Interleukin-6
IL-1*β*: Interleukin-1 beta
NF-*κ*B: Nuclear factor-kappa B
MAPK: Mitogen-activated protein kinases
AST: Aspartate aminotransferase
ALT: Alanine transaminase
ADH: Alcohol dehydrogenase
MPO: Myeloperoxidase
ALDH: Acetaldehyde dehydrogenase
GSSG: Glutathione disulfide
ATP: Adenosine triphosphate
PXR: Pregnane X receptor
PGE2: Prostaglandin E2.
